# Supplementary material for: Gene gain and loss push prokaryotes beyond the homologous recombination barrier and accelerate genome sequence divergence
Source: Nat Commun. 2019 Nov 26;10:5376. doi: 10.1038/s41467-019-13429-2 (PMC6879757; doi:10.1038/s41467-019-13429-2)
Supplement: Supplementary file 1 — Supplementary Information [file 41467_2019_13429_MOESM1_ESM.pdf]

Supplementary Information:

Gene gain and loss push prokaryotes beyond the homologous recombination barrier and accelerate genome sequence divergence

Iranzo et al.

Contents

Supplementary Figures 2
Supplementary Figure 1 . . . 2
Supplementary Figure 2 . . . 3
Supplementary Figure 3 . . . 4
Supplementary Figure 4 . . . 6
Supplementary Figure 5 . . . 8
Supplementary Figure 6 . . . 9
Supplementary Figure 7 . . . 10

Supplementary Note 1: Genome content divergence 11

Supplementary Note 2: Delay in sequence divergence driven by homologous recombination 12

Supplementary Note 3: Analytical study of simple cases 13
Case $R = \lambda t^\gamma$ . . . 14
Case $R = \lambda_0 + \lambda_1 t$ . . . 14
Case $R = \lambda_0 - \lambda_1 f$ . . . 15

Supplementary Note 4: Comparison of sequence and genome content divergences 15

Supplementary Note 5: Dispersion of evolutionary rates 16

Supplementary References 17

Supplementary Figures

Supplementary Figure 1

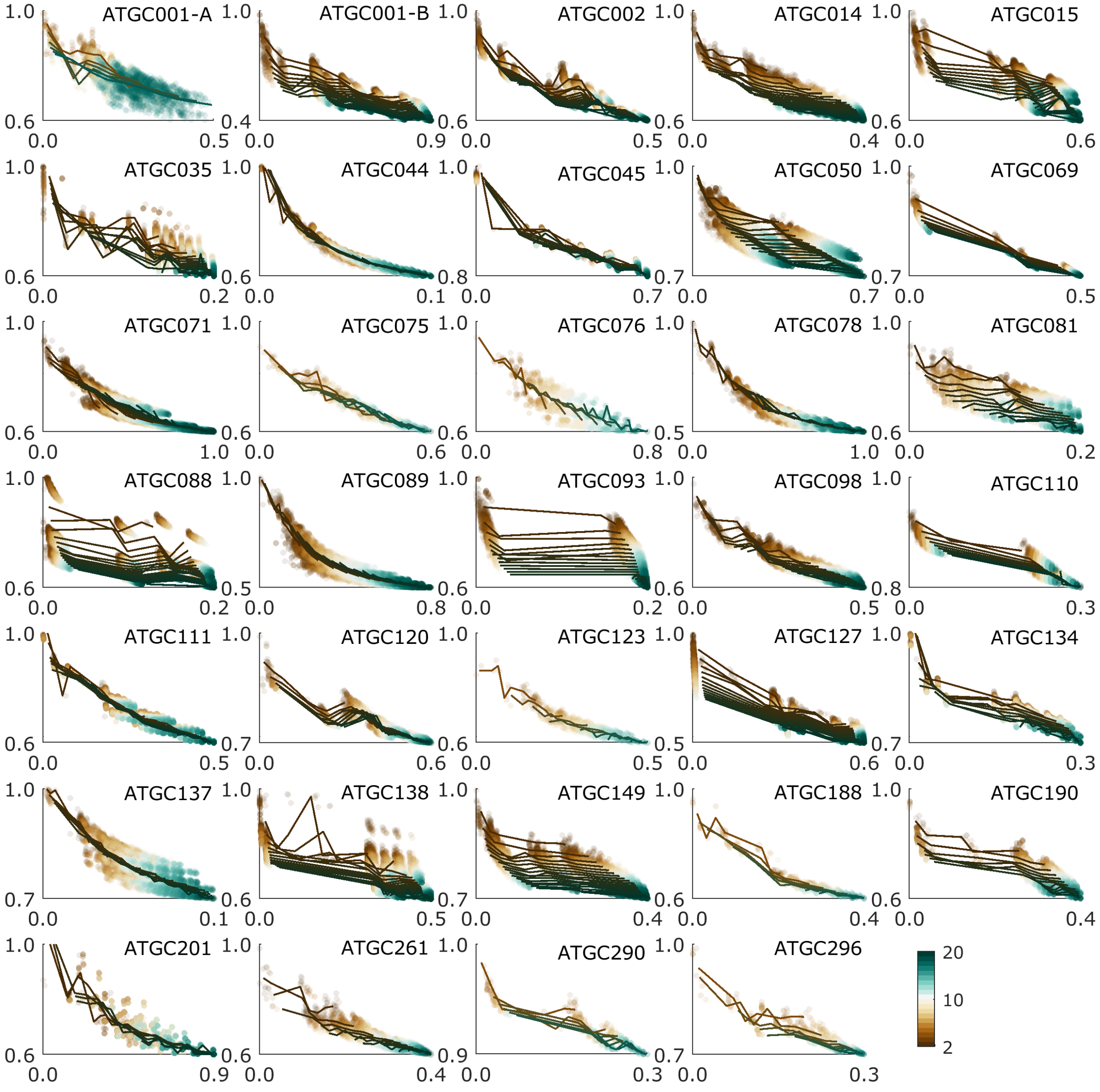

Supplementary Figure 1. Fraction of shared genes as a function of the total tree distance, based on traditional sequence similarity trees. Colors indicate the number of genomes sampled in each case. Numeric labels in all axes have been rounded to the closest first decimal. Source data are provided as a Source Data file.

Supplementary Figure 2

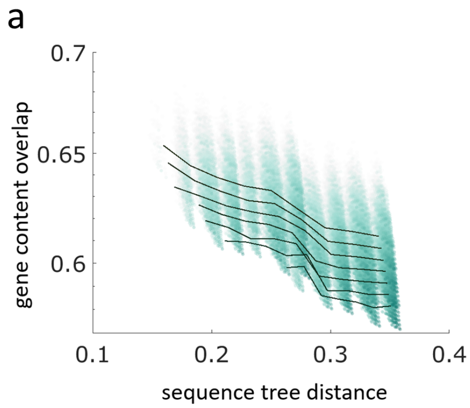

Supplementary Figure 2. Gene content decay curves of the *Bacillus thuringiensis/cereus/anthracis* group for genes that are present in 10 genomes or more. a) The gene content decay curves shift down as the number of sampled genomes increases, which is compatible with a delayed onset of the sequence-based molecular clock with respect to the gene gain and loss clock. The figure is based on widespread genes found in 10 genomes or more, which are most likely important for the respective organisms and evolve slowly. Consequently, their presence does not represent recent acquisition events. b) When the sequence-based phylogenetic tree is rescaled according to the recombination model to compensate for the delay in the molecular clock, the expected overlap among gene content decay curves is recovered. Source data are provided as a Source Data file.

Supplementary Figure 3

Supplementary Figure 3 ***(following page).*** Recent gene acquisitions, associated with gene families that are gained and lost at high rates, do not bias the estimation of gene content distances used in our study. We simulated genome evolution through gene gain and loss over a phylogeny with topology and branching times representative of those observed in the ATGC dataset. The simulations included two classes of genes with different gain and loss rates and assumed an infinite gene pool. To ensure that our simulations had produced populations with an excess of recent, non-fixed gene gains and losses, we calculated the fraction of differences in gene content per simulated time (a). As expected, the gain and loss of fast-turnover genes that will not reach fixation in the long term led to a faster accumulation of genomic differences in pairs of genomes that diverged towards the end of the simulation. To assess the effect of such gains and losses on our analysis, we processed the simulated genomes as we did with the real dataset, applying a phylogenomic reconstruction tool (Gloome [1]) to infer gene content trees, while controlling for family-specific gene gain and loss rates. We found that the branch lengths of the inferred gene content trees were proportional to the simulated time intervals; in particular, the excess of genomic differences observed in (a) did not lead to the overestimation of the lengths of shallow branches in the gene content trees (b). Accordingly, the branch lengths of the inferred trees represent the expected number of gene gains and losses that reach fixation along the branch. (c) In blue, the simulation results confirm that gene content distances are proportional to the (simulated) evolutionary time, as long as they are defined as the sum of branch lengths between two genomes in the inferred gene content tree (as we did in the analysis of the real dataset). Importantly, that would not be the case if gene content distances were naively defined as the fraction of different genes between a pair of genomes (in orange, $D = -\log(1-p)$, where p is the fraction of genes that differ between the two genomes); with such simple definition (which we did not use for our analysis), recent gain and loss events would artificially increase gene content distances between genomes.

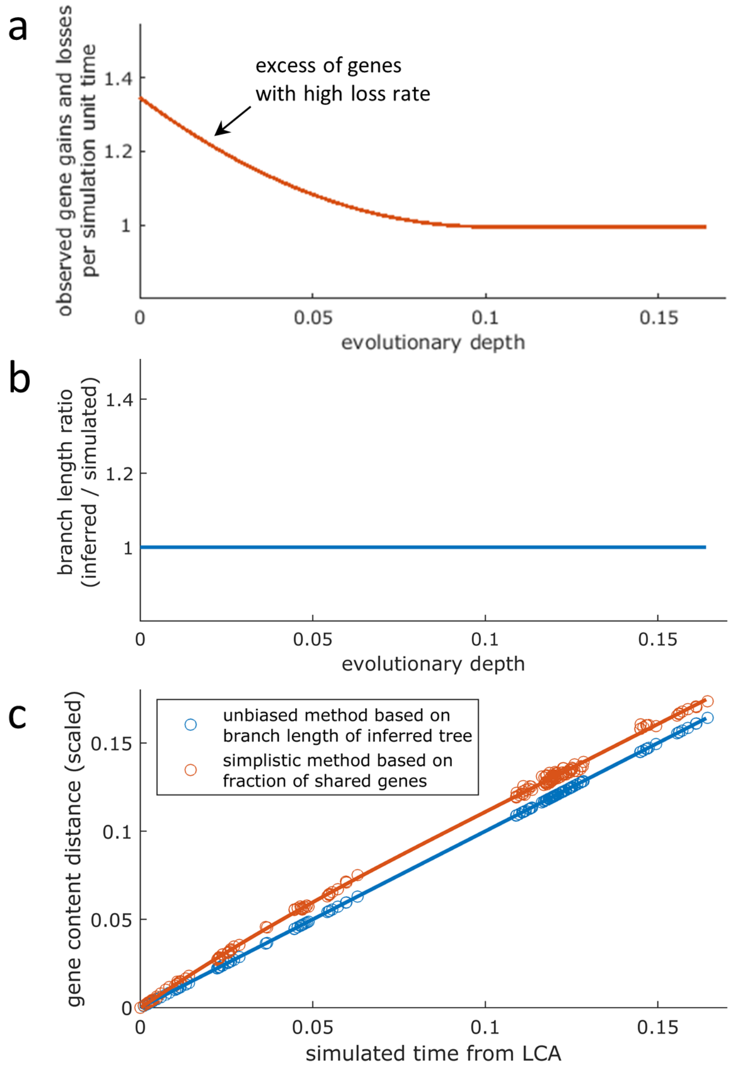
a
observed gene gains and losses per simulation unit time
excess of genes with high loss rate
1.4
1.2
1
0
0.05
0.1
0.15
evolutionary depth
b
branch length ratio (inferred / simulated)
1.4
1.2
1
0
0.05
0.1
0.15
evolutionary depth
c
gene content distance (scaled)
unbiased method based on branch length of inferred tree
simplistic method based on fraction of shared genes
0.15
0.1
0.05
0
0
0.05
0.1
0.15
simulated time from LCA

Supplementary Figure 4

Supplementary Figure 4 *(following page).* Recent gene acquisitions, associated with neutral and deleterious gene families that do not reach fixation in the long term, do not bias the estimation of gene content distances used in our study. We simulated genome evolution through gene gain and loss as a Moran process in a population of $N = 10^4$ genomes that split following a realistic phylogenetic tree. At each branching point, one genome was randomly chosen with probability proportional to its fitness, and that genome was used as the clonal seed for the two daughter populations. Because of these bottlenecks, the effective population size was $N_e \approx \sqrt{N} = 10^2$. Two classes of genes were considered: (i) a neutral class, and (ii) a deleterious class with $s = -0.02$. Because $|N_e s| > 1$, genes of the second class are rapidly eliminated by selection and only persist in the genome for short evolutionary times. Gain and loss rates were adjusted to obtain an average of 1000 neutral genes and 2 (a-c) or 5 (d-f) deleterious genes per genome. To ensure that our simulations had produced populations with an excess of recent, non-fixed gene gains and losses, we calculated the fraction of differences in gene content per simulated time (a,d). As expected, the acquisition of neutral and deleterious genes that will not reach fixation in the long term led to a faster accumulation of genomic differences in pairs of genomes that diverged towards the end of the simulation. To assess the effect of recent, non-fixed gene gains and losses on our analysis, we processed the simulated genomes as we did with the real dataset, applying a phylogenomic reconstruction tool (Gloome [1]) to infer gene content trees, while controlling for family-specific gene gain and loss rates. We found that the branch lengths of the inferred gene content trees were proportional to the simulated time intervals; in particular, the excess of genomic differences observed in (a,d) did not lead to the overestimation of the lengths of shallow branches in the gene content trees (b,e). Accordingly, the branch lengths of the inferred trees represent the expected number of gene gains and losses that reach fixation along the branch. (c,f) In blue, the simulation results confirm that gene content distances are proportional to the (simulated) evolutionary time, as long as they are defined as the sum of branch lengths between two genomes in the inferred gene content tree (as we did in the analysis of the real dataset). Importantly, that would not be the case if gene content distances were naively defined as the fraction of different genes between a pair of genomes (in orange, $D = -\log(1-p)$, where p is the fraction of genes that differ between the two genomes); with such simple definition (which we did not use for our analysis), recent gain of neutral and deleterious genes would artificially increase gene content distances between genomes.

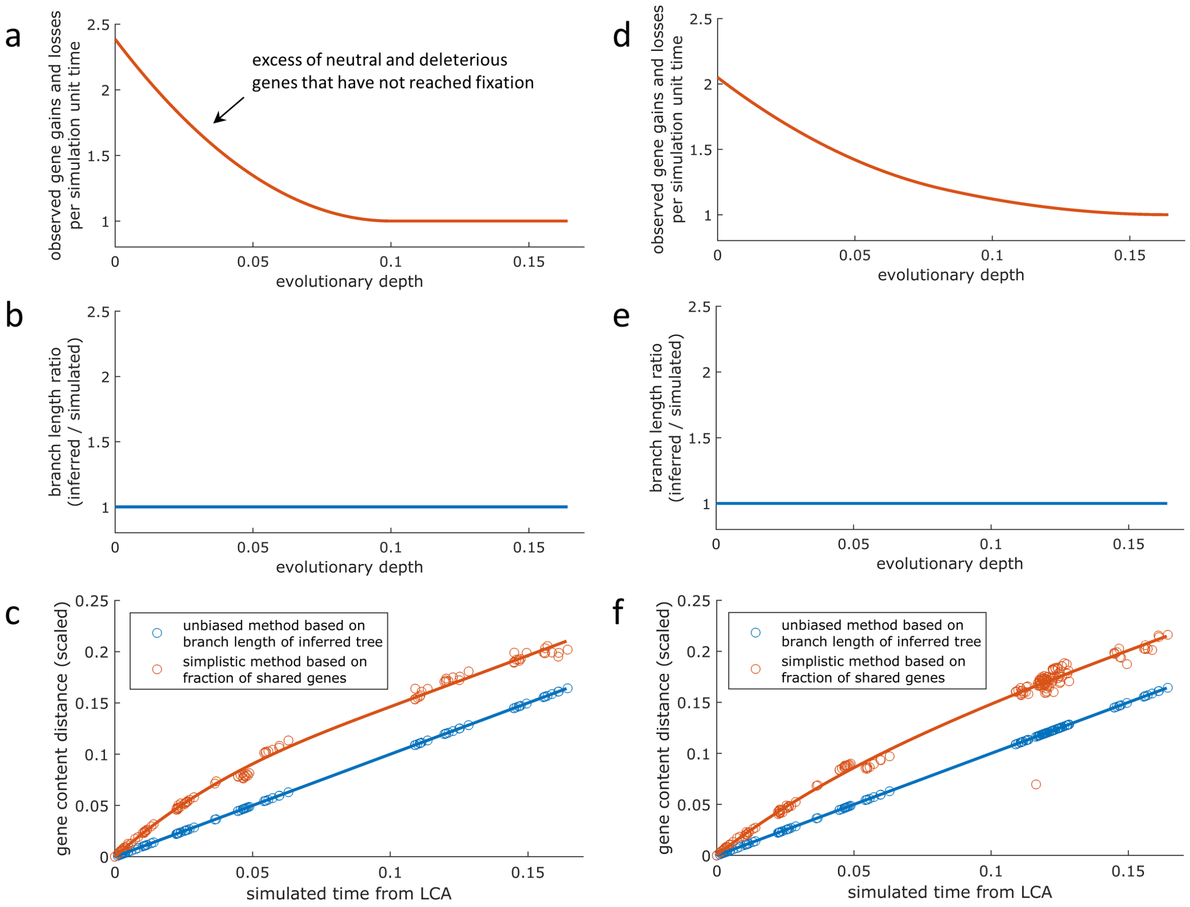
a
observed gene gains and losses per simulation unit time
excess of neutral and deleterious genes that have not reached fixation
evolutionary depth
b
branch length ratio (inferred / simulated)
evolutionary depth
c
gene content distance (scaled)
unbiased method based on branch length of inferred tree
simplistic method based on fraction of shared genes
simulated time from LCA
d
observed gene gains and losses per simulation unit time
evolutionary depth
e
branch length ratio (inferred / simulated)
evolutionary depth
f
gene content distance (scaled)
unbiased method based on branch length of inferred tree
simplistic method based on fraction of shared genes
simulated time from LCA

Supplementary Figure 5

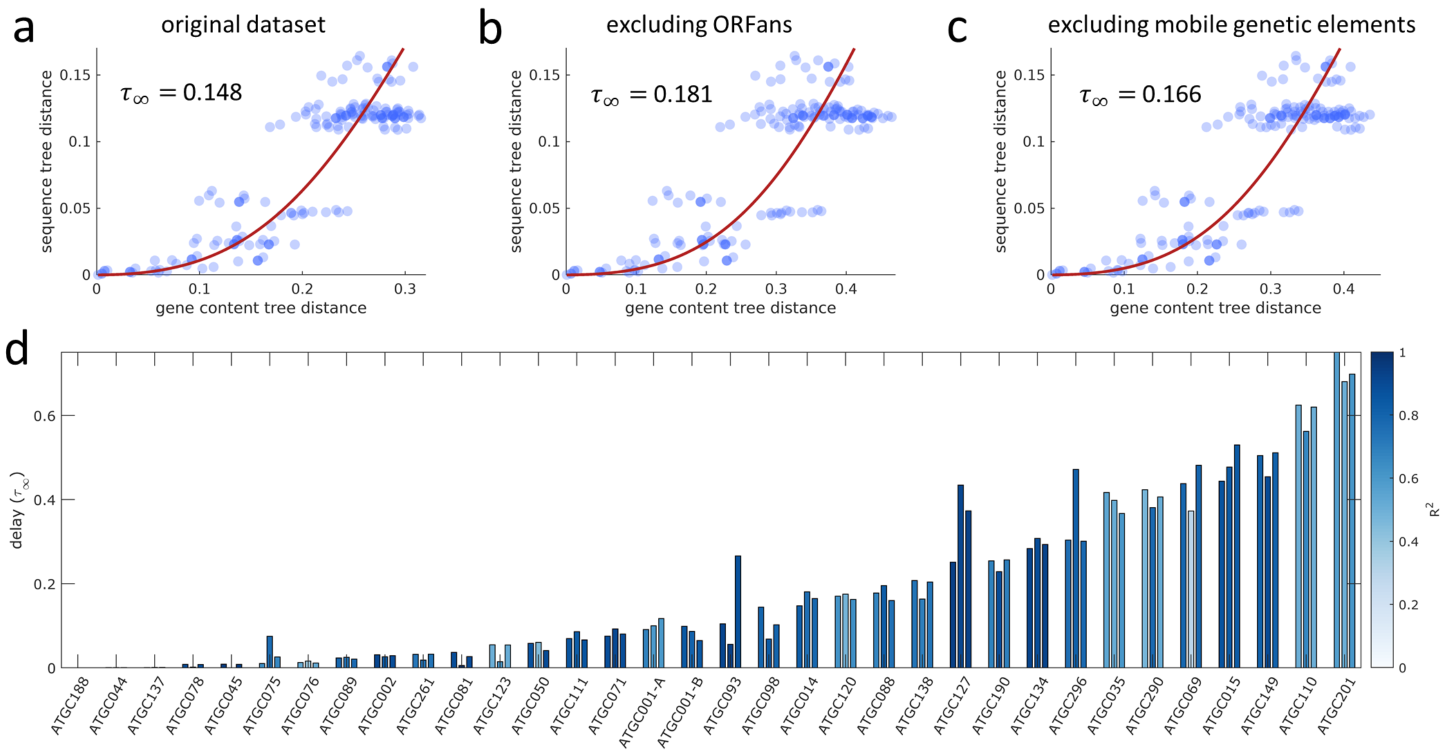

Supplementary Figure 5. Evolutionary delays in the ATGCs after removing potentially deleterious and rare neutral genes, which are unlikely to reach fixation in the long term. To verify that the estimates for the delay time are not affected by recent gene acquisition or deletion events, we compared the delay times inferred by using all ATGC-COGs (a), all genes but bona fide ORFans (genes found in a single genome, with no orthologs in the ATGC database) (b), and ATGC-COGs that do not belong to mobile genetic elements (c). Scatter plots in panels (a-c) show the comparison of pairwise distances between leaves in the sequence-based phylogenetic tree and the gene content tree of ATGC014 (*Bacillus thuringiensis/cereus/anthracis* group); red lines show the fit of the data to the recombination barrier model. (d) Estimated delays for all ATGCs when the analysis is performed in (left to right) all ATGC COGs, all genes but bona fide ORFans, and ATGC COGs that do not belong to mobile genetic elements. The bar color is indicative of the goodness of fit. Source data are provided as a Source Data file.

Supplementary Figure 6

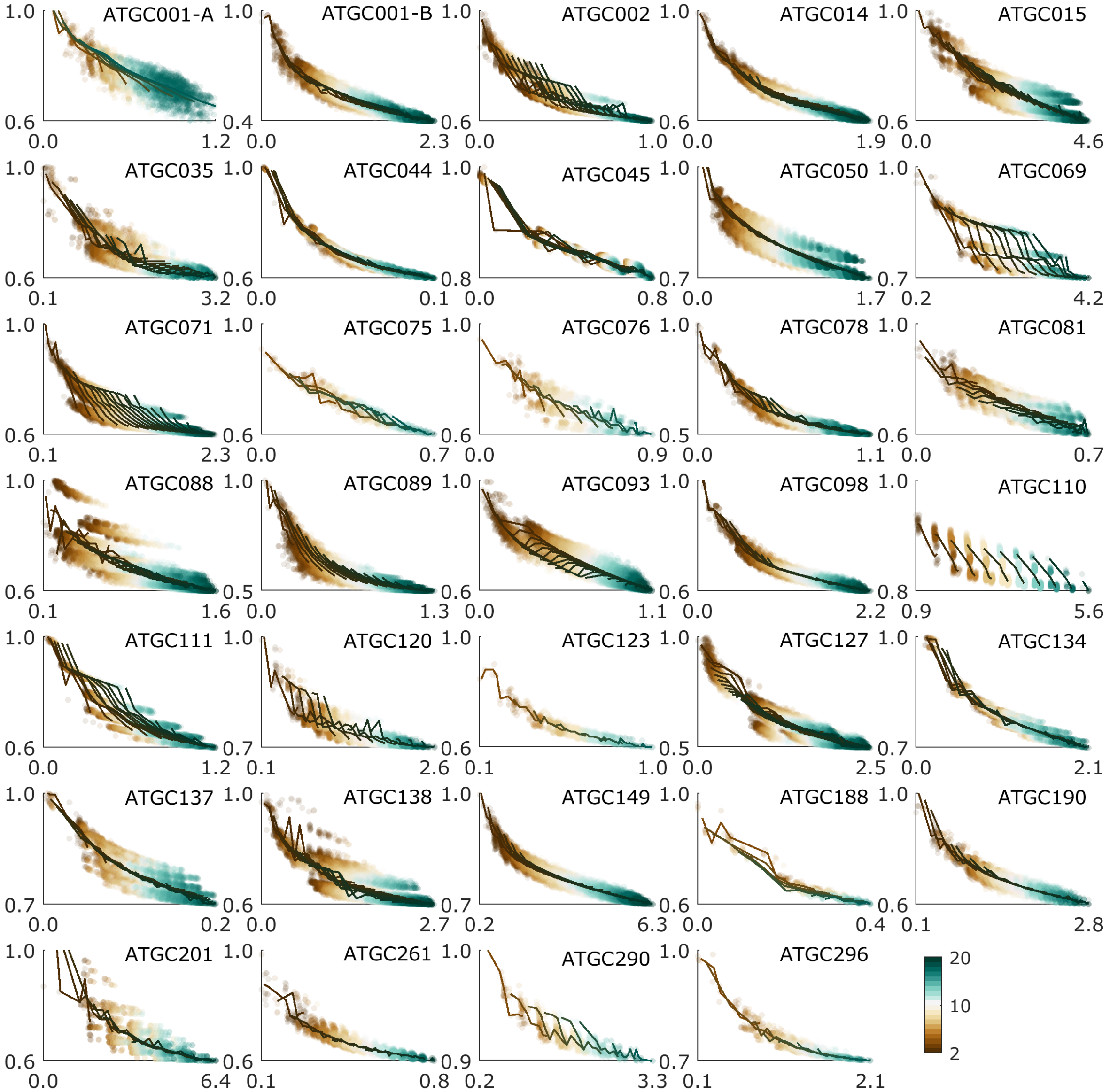

Supplementary Figure 6. Fraction of shared genes as a function of the total tree distance, based on phylogenetic trees whose branch lengths have been corrected to account for the recombination-driven delay in the molecular clock. The corrected branch lengths were obtained by applying the recombination barrier model with the ATGC-specific parameters from Supplementary Table 1 (see Methods). Colors indicate the number of genomes sampled in each case. Numeric labels in all axes have been rounded to the closest first decimal. Source data are provided as a Source Data file.

Supplementary Figure 7

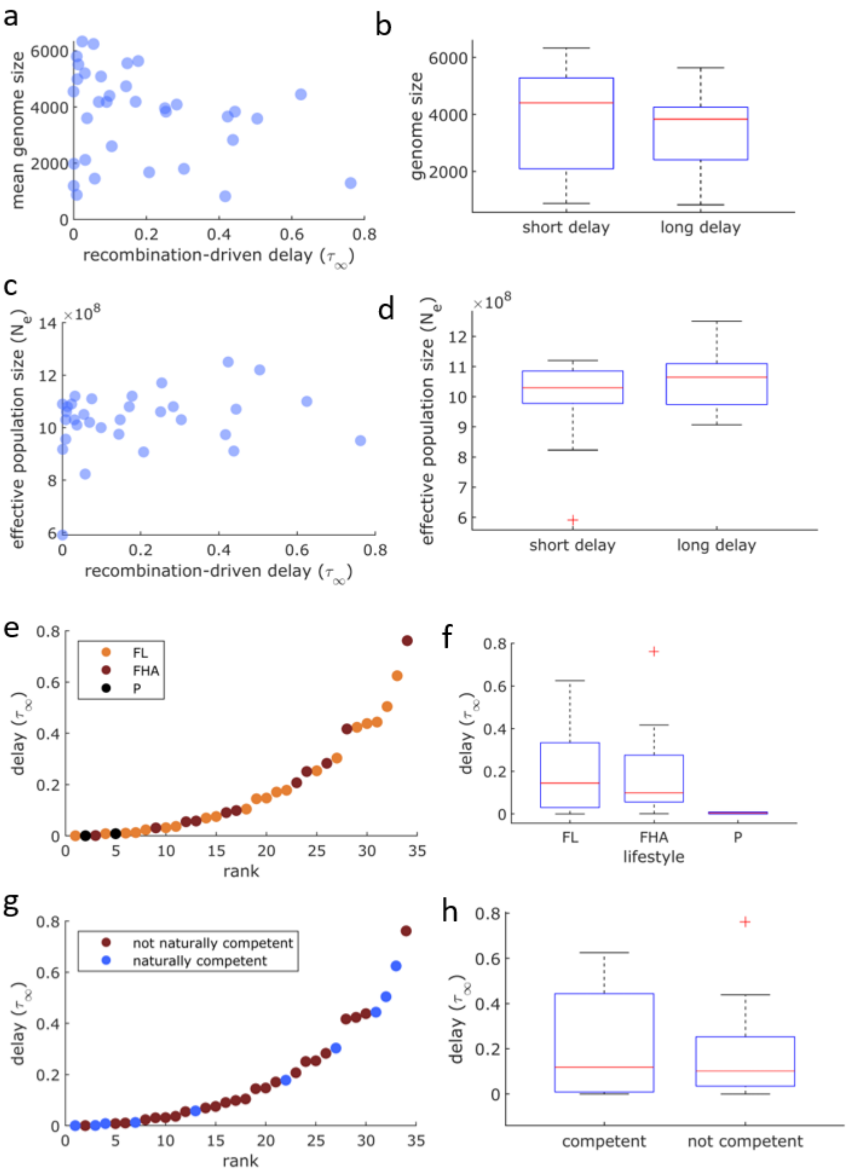

Supplementary Figure 7. Genomic and ecological variables that do not show significant statistical association with the recombination-driven delay (τ_∞). Each data point corresponds to one ATGC. (a,b) Mean genome size, measured as number of genes; (c,d) effective population size, calculated as described in [2]; (e,f) life style, classified as free-living (FL), facultative host-associated (FHA), and obligate parasite (P); (g,h) natural competence for transformation. Short and long delays refer to the relative magnitude of the delay with respect with the total tree depth. Source data are provided as a Source Data file.

Supplementary Note 1: Genome content divergence

The number of genes x evolves under stochastic dynamics that is given by the equation

$$\dot{x} = P_+ - P_- \tag{1}$$

where P_+ and P_- denote gain and loss rates of one gene, respectively. The intersection of k genomes I_k is given by

$$I_k = \langle \text{Intersect}\,(\mathbf{X}_1 \ldots \mathbf{X}_k) \rangle \tag{2}$$

where X_i is an array representation of a genome that assumes values of 0 or 1 in each entry, indicating presence or absence of genes in each genome. Accordingly, the dynamics of the intersection is given by

$$\dot{I}_k = k\langle \text{Intersect}(\dot{\mathbf{X}}_1 \ldots \mathbf{X}_k) \rangle \tag{3}$$

where the angled brakets indicate averaging on many realizations, and we used the fact that

$$\langle \text{Intersect}(\ldots \dot{\mathbf{X}}_n, \mathbf{X}_m \ldots) \rangle = \langle \text{Intersect}(\ldots \mathbf{X}_n, \dot{\mathbf{X}}_m \ldots) \rangle \tag{4}$$

Assuming an infinite gene pool, intersections evolve due to loss events, where every time a gene is lost there is a probablity that the lost gene belongs to the intersection, such that

$$\langle \text{Intersect}(\dot{\mathbf{X}}_1 \ldots \mathbf{X}_k) \rangle = -P_- I_k / x \tag{5}$$

Substituting Eq. (5) into Eq. (3), we get

$$\dot{I}_k = -kP_- I_k / x \tag{6}$$

For constant gain and loss rates, the solution of the above equation is given by

$$I_k(t) = I_k(0) \cdot \left[1 + \frac{P_+ - P_-}{x} \cdot t \right]^{-\frac{kP_-}{P_+ - P_-}} \tag{7}$$

In the limit of equilibrium genome size, $(P_+ - P_-) \to 0$, the intersection decays exponentially

$$I_k(t) = I_k(0) \cdot e^{-(kP_-/x)t} \tag{8}$$

For two genomes, the intersection at $t = 0$ is given by the mean genome size x and we have

$$I_2(t) = x \cdot e^{-(\nu)T_2} \tag{9}$$

where the decay constant is given by $\nu = 2P_-/x$ and T_2 is the time since the last common ancestor of the two intersecting genomes. For three genomes, the general result of Eq. (8) takes the form

$$I_3(t) = I_3(0) \cdot e^{-(3P_-/x)T_3} \tag{10}$$

where T_3 measures the time of divergence of three genomes (see Supplementary Fig. 8). Accordingly, $I_3(0)$ is the number of shared genes in two genomes right before the branching event that produced the third genome:

$$I_3(0) = x \cdot e^{-(2P_-/x)T_2} \tag{11}$$

Substituting Eq. (11) into Eq. (10) we get

$$I_3(t) = x \cdot e^{-(P_-/x)\tau_3} \tag{12}$$

where $\tau_3 = 2T_2 + 3T_3$ is the total evolution time of the three diverging genomes. Assuming a clock, that is, that tree branch lengths are proportional to the time variable, Eq. (12) can be written in terms of tree distances

$$I_3(t) = x \cdot e^{-\lambda D_3} \tag{13}$$

where $\lambda \propto P_-/x$ and $D_3 = \sum d_i$ is the sum of all branch lengths in the tree spanned by the three intersecting genomes. The above result for three intersecting genomes can be generalized to k genomes, leading to the expression

$$I_k(t) = x \cdot e^{-\lambda D_k} \tag{14}$$

Similarly, if there are multiple classes of genes that differ in their loss rates, with $P_{-,i}$ denoting the loss rate of genes from class i, the number of genes shared by k genomes becomes

$$I_k(t) = \sum_i x_i \cdot e^{-\lambda_i D_k} \tag{15}$$

where x_i is the mean abundance of genes from class i and $\lambda_i \propto P_{-,i}/x_i$.

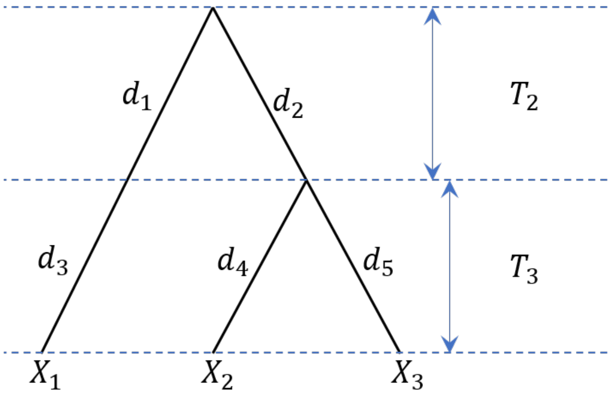

Supplementary Figure 8. Phylogenetic tree depicting divergence of three genomes, denoted as X_1, X_2 and X_3. Branch lengths are denoted by d_i, and branching times are indicated by dashed blue lines. Initially, two genomes diverge for time T_2. Following a branching event at T_2, three genomes diverge for time T_3.

Supplementary Note 2: Delay in sequence divergence driven by homologous recombination

Previous modeling and comparative genomic studies have shown that mutation accumulation in the presence of homologous recombination is characterized by "recombination barriers", such that once a genomic region diverges beyond the recombination barrier, it is unlikely to experience further homologous recombination with members of the ancestral population. The escape from the recombination barrier is a stochastic process that depends on molecular and ecological details, such as the specific mechanism of recombination, the dependency of the recombination efficiency on sequence conservation, and the effective population size. Here we take a phenomenological approach that allows us to model the dynamics of sequence divergence without making specific assumptions about the mechanisms that underlie the existence of recombination barriers. We start by defining the fraction of the genome that is subject to

recombination as f. As regions of the genome escape the recombination barrier, the fraction subject to homologous recombination is

$$\frac{\mathrm{d}f}{\mathrm{d}t} = -Rf \tag{16}$$

where R is the rate at which regions of the genome escape the recombination barrier. R can be a constant (as suggested by [3]) or any arbitrary function of t and f. The probability that a region of the genome escapes exactly at time t is

$$P(t) = -\frac{\mathrm{d}f}{\mathrm{d}t} \tag{17}$$

Although substitutions can occur anywhere in the genome, regions subject to homologous recombination periodically revert to the original sequence and therefore their contribution to the overall sequence divergence is minor. Instead, we focus on the accumulation of substitutions in regions that escape the recombination barrier, which occurs at a rate μ. For a region that escaped the recombination barrier at time u, its contribution to the overall sequence divergence is equal to $\mu(t-u)$. Integrating over all possible escape times, we obtain the following expression for the genome-level sequence divergence between two genomes:

$$\frac{\Delta(t)}{2\mu} = \int_0^t (t-u)\,P(u)\,\mathrm{d}u \tag{18}$$

Integration by parts leads to

$$\frac{\Delta(t)}{2\mu} = -tF(0) + \int_0^t F(u)\,\mathrm{d}u \tag{19}$$

where $F(u) = \int_0^u P(w)\mathrm{d}w$. It follows from Eq. (17) that $F(u) = -f(u)$. Moreover, assuming an initial state in which the whole genome is subject to homologous recombination, $f(0) = 1$, the overall sequence divergence becomes

$$\Delta(t) = 2\mu\left(t - \int_0^t f(u)\mathrm{d}u\right) \tag{20}$$

The integral term defines the delay in the molecular clock driven by homologous recombination.

Equation (20) captures the effect of homologous recombination on sequence divergence by assuming that gene conversion resets divergence to zero. In reality, recombination homogenizes the population by making the distance between two recombining loci independent of their evolutionary history. If recombination is strong, the contribution of recombining loci to pairwise sequence divergence is approximately equal to the population diversity θ [3]. Thus, the general expression for pairwise sequence divergence becomes

$$\Delta(t) = 2\mu\left(t - \int_0^t f(u)\mathrm{d}u\right) + \tilde{\theta} \tag{21}$$

where $0 \leq \tilde{\theta} \lesssim \theta$ (and $\theta = 2\mu N_e$ in the limit of neutral evolution).

Supplementary Note 3: Analytical study of simple cases

In general, the delay term in Eq. (20) depends on the dynamics of the escape from the recombination barrier which itself depends on molecular and ecological factors, such as the recombination mechanisms and the effective population size. From a purely phenomenological perspective, exact expressions for the delay term can be obtained by considering possible functional dependencies of the escape rate on the time since the last common ancestor and the fraction of escaped regions.

Case $R = \lambda t^\gamma$

Here we consider the general situation where the escape rate is proportional to an arbitrary power of the time variable. This includes the particular case of a constant escape rate, as well as cases where the escape rate accelerates or decelerates with time. The differential equation for the fraction of the genome subject to homologous recombination is

$$\frac{\mathrm{d}f}{f} = -\lambda t^\gamma \, \mathrm{d}t \tag{22}$$

with the boundary condition $f(0) = 1$. The boundary condition can only be satisfied if $\gamma > 1$, in which case we get

$$f(t) = \exp\left(-\frac{\lambda\, t^{\gamma+1}}{\gamma+1}\right) \tag{23}$$

The delay term in Eq. (20) is obtained by integrating the function f. Simple analytical expressions are obtained in the special cases $\gamma = 0$ (constant escape rate) and $\gamma = 1$ (linear growth of the escape rate with time).

$$\gamma = 0 \quad \Rightarrow \quad \frac{\Delta(t)}{\mu} = t - \tau\,(1 - e^{-t/\tau}) \quad \text{with} \quad \tau = 1/\lambda \tag{24}$$

$$\gamma = 1 \quad \Rightarrow \quad \frac{\Delta(t)}{\mu} = t - \tau \ \mathrm{erf}\left(\frac{\sqrt{\pi}}{2}\frac{t}{\tau}\right) \quad \text{with} \quad \tau = \sqrt{\frac{\pi}{2\lambda}} \tag{25}$$

where erf is the error function. In these expressions, $\tau_\infty = \tau$ is the long-term delay in the molecular clock induced by homologous recombination.

Case $R = \lambda_0 + \lambda_1 t$

When the escape rate includes a constant term plus a linear term in the time variable, the fraction of the genome susceptible to homologous recombination follows the differential equation

$$\frac{\mathrm{d}f}{f} = -(\lambda_0 + \lambda_1 t)\,\mathrm{d}t \tag{26}$$

with solution

$$f(t) = \exp\left(-t\left(\lambda_0 + \frac{\lambda_1}{2}\,t\right)\right) \tag{27}$$

Substituting Eq. (27) into Eq. (20), the overall sequence divergence becomes

$$\frac{\Delta(t)}{2\mu} = t - \int_0^t e^{-u\,\left(\lambda_0 + \frac{\lambda_1}{2}\,u\right)}\,\mathrm{d}u \tag{28}$$

$$= t - \tau_1\, e^{\frac{1}{\pi}\left(\frac{\tau_1}{\tau_0}\right)^2}\left[\mathrm{erf}\left(\frac{1}{\sqrt{\pi}}\frac{\tau_1}{\tau_0} + \frac{\sqrt{\pi}}{2}\frac{t}{\tau_1}\right) - \mathrm{erf}\left(\frac{1}{\sqrt{\pi}}\frac{\tau_1}{\tau_0}\right)\right] \tag{29}$$

where $\tau_0 = \frac{1}{\lambda_0}$ and $\tau_1 = \sqrt{\frac{\pi}{2\lambda_1}}$. In the limit $t \to \infty$, the long-term delay in the molecular clock becomes

$$\tau_\infty = \tau_1\, e^{\frac{1}{\pi}\left(\frac{\tau_1}{\tau_0}\right)^2}\left[1 - \mathrm{erf}\left(\frac{1}{\sqrt{\pi}}\frac{\tau_1}{\tau_0}\right)\right] \tag{30}$$

Case $R = \lambda_0 - \lambda_1 f$

This case covers the more realistic scenario in which the escape rate depends on the fraction of the genome that has already crossed the recombination barrier. Intuitively, the probability that a region of interest is surrounded by well conserved regions that can act as seeds for homologous recombination will depend on how many of such well conserved regions still exist in the genome. As a result, the rate at which genomic regions escape the recombination barrier will accelerate as the fraction of regions that are still subject to recombination decreases. A simple model for this autocatalytic scenario is represented by the following differential equation:

$$\frac{\mathrm{d}f}{\mathrm{d}t} = -\lambda_0 f + \lambda_1 f^2 \tag{31}$$

For consistency with the biological interpretation, we impose the constraint $\lambda_0 > \lambda_1 > 0$. The solution of this equation with the boundary condition $f(0) = 1$ is

$$f(t) = \frac{1 + e^{-\phi}}{1 + e^{-\phi(1-t/\tau)}} \tag{32}$$

where $\phi = \ln\left(\frac{\lambda_1}{\lambda_0 - \lambda_1}\right)$ and $\tau = \phi/\lambda_0$. The expression in Eq. (32) describes a sigmoidal dynamics with a transition from $f = 1$ (homologous recombination affecting the entire genome) to $f = 0$ (total escape from the pull of recombination) around a characteristic time τ. Parameter ϕ describes the steepness of the transition, with greater values of ϕ implying a faster transition. The substitution of Eq. (32) into Eq. (20) provides the following expression for the overall sequence divergence:

$$\frac{\Delta(t)}{2\mu} = t - \tau\,\frac{1 + e^{-\phi}}{\phi}\,\ln\left(\frac{1 + e^{\phi}}{1 + e^{\phi(1-t/\tau)}}\right) \tag{33}$$

In this case, the long-term delay in the molecular clock becomes

$$\tau_\infty = \tau\,(1 + e^{-\phi})\,\ln(1 + e^{\phi})/\phi = \ln\left(\frac{\lambda_0}{\lambda_0 - \lambda_1}\right)/\lambda_1 \tag{34}$$

Supplementary Note 4: Comparison of sequence and genome content divergences

Let us consider a gene turnover clock with rate λ. Then, the pairwise divergence in the genome content at time t after the last common ancestor can be expressed as

$$\Delta_G = 2\lambda t \tag{35}$$

If all genes have the same turnover rate, the divergence can be directly inferred from Eq. (14) by taking $\Delta_G = -\frac{1}{2}\log(1 - J)$, where $J = (x - I_2)/x$ is the Jaccard distance, i.e. the fraction of genes that differ between the two genomes. If gene turnover rates are family-specific, this formula overestimates the divergence between closely related genomes, thereby producing the illusion of a non-linear clock (see Supplementary Fig. 3). The reason is that fast turnover genes, that are quickly gained and lost, contribute to the divergence of very closely related genomes to a greater extent than they do for distant genomes. Therefore, to avoid biases, it is essential that estimates of genome divergence take into account the heterogeneity of gene turnover rates. In this study, such estimates were obtained by running the phylogenomic reconstruction program Gloome [1] to optimize the branch lengths of genome content trees under a genome evolution model with 4 categories of gamma-distributed gain and loss rates.

Previous theoretical and modeling work has suggested that controlling for family-specific gene gain and loss rates could also prevent biases associated with family-specific fitness effects, including those due

to the presence of deleterious genes that are transiently acquired (but not fixed) in the terminal branches of the phylogenetic tree [4]. To validate this, we simulated a population of genomes composed of neutral and deleterious genes. We found that phylogenomic reconstruction under the assumptions of neutrality and family-specific gain and loss rates produced genome content trees with branches scaling linearly with the simulated time (see Supplementary Fig. 4).

The divergence at the levels of sequence and genome content can be compared by combining Eq. (35) with the equation for pairwise sequence divergence, $\Delta_s = 2\mu(t - \tau(t)) + \tilde{\theta}$ (Eq. 21)

$$\frac{\Delta_s}{\mu} = \frac{\Delta_G}{\lambda} - \tau(t) + \frac{\tilde{\theta}}{\mu} \tag{36}$$

Because $\tau(t)$ is a saturating function, at sufficiently long times the relationship between sequence and gene content divergence will reach a linear regime

$$\lim_{t\to\infty} \Delta_s = a\Delta_G + b \tag{37}$$

The slope of the linear regime, $a = \mu/\lambda$, is determined by the relative rate of substitutions with respect to gene turnover; the intercept, $b = -\mu\tau_\infty + \tilde{\theta}$ provides a lower bound for the long-term recombination driven delay, such that $\tau_\infty \geq -b/\mu$.

Supplementary Note 5: Dispersion of evolutionary rates

In Supplementary Note 2, we focused on the genome-level sequence divergence which results from different regions escaping the recombination barrier at different times. In this Supplementary Note, we study the rates at which individual regions accumulate mutations. Let $\Pr(D|t)$ be the probability that the sequence divergence in the region of interest is equal to D at time t. Assuming that the main source of stochasticity in the divergence process is the time required to escape the recombination barrier, we can write

$$\Pr(D|t) = \begin{cases} 1 - \int_0^t P(u)\mathrm{d}u & \text{if } D = 0 \\ P\left(t - D/\mu\right)/\mu & \text{if } 0 < D \leq \mu t \\ 0 & \text{if } D > \mu t \end{cases} \tag{38}$$

where $P(u)$ is the probability that the region escapes the recombination barrier exactly at time u.

Let us now introduce the 'genome-relative evolutionary rate', r, as the ratio between the divergence of a region and the genome-level divergence at a given time:

$$r = \frac{D}{\langle D|t\rangle_G} \tag{39}$$

The angle brackets $\langle\cdot\rangle_G$ indicate the average within a genome. Such averaging affects both the escape times and the substitution rates, because substitution rates can vary across regions. Assuming that the escape times are independent of the substitution rates and applying Eq. (18), we have

$$\langle D|t\rangle_G = \langle\mu\rangle_G \int_0^t (t-u)P(u)\mathrm{d}u \tag{40}$$

In closely related genomes with a high degree of synteny, the variability in substitution rates across regions can be accounted for by normalizing by the average evolutionary rates of the genes that occupy each region. To this end, we define the 'relative evolutionary rate' as:

$$\delta = \frac{r}{\langle r|t\rangle_L} = \frac{D}{\langle D|t\rangle_G \, \langle r|t\rangle_L} \tag{41}$$

where $\langle\cdot\rangle_L$ denotes the average for a specific locus across closely related genomes. Note that the relative evolutionary rate defined in this way controls for basal differences in the substitution rate across genes. The value of $\langle r|t\rangle_L$ can be calculated by taking the average in Eq. (39), assuming a constant value of μ for the locus of interest:

$$\langle r|t\rangle_L = \frac{\langle r|t\rangle_L}{\langle D|t\rangle_G} = \frac{\mu}{\langle\mu\rangle_G} \tag{42}$$

and combining Eq. (41) and (42) we get to the final expression for the relative evolutionary rate:

$$\delta = \frac{D\,\langle\mu\rangle_G}{\mu\,\langle D|t\rangle_G} \tag{43}$$

Let $\Pr(\delta|t)$ be the probability that the relative evolutionary rate of a genomic region is equal to δ at time t. An explicit expression for $\Pr(\delta|t)$ can be obtained by substituting Eq. (43)in Eq. (38):

$$\Pr(\delta|t) = \begin{cases} 1 - \int_0^t P(u)\mathrm{d}u & \text{if } \delta = 0 \\ P\left(t - \frac{\delta\langle D|t\rangle_G}{\langle\mu\rangle_G}\right)\frac{\langle D|t\rangle_G}{\langle\mu\rangle_G} & \text{if } 0 < \delta \le \frac{t\,\langle\mu\rangle_G}{\langle D|t\rangle_G} \\ 0 & \text{if } \delta > \frac{t\,\langle\mu\rangle_G}{\langle D|t\rangle_G} \end{cases} \tag{44}$$

The genomic variance of δ at a given time is calculated as $\mathrm{Var}(\delta|t) = \langle(\delta|t)^2\rangle_G - \langle\delta|t\rangle_G^2$. The quadratic mean is equal to

$$\langle(\delta|t)^2\rangle_G = \int_0^{\frac{t\,\langle\mu\rangle_G}{\langle D|t\rangle_G}} \delta^2\,P(\delta|t)\,\mathrm{d}\delta = \frac{\langle\mu\rangle_G^2}{\langle D|t\rangle_G^2}\int_0^t (t-u)^2 P(u)\mathrm{d}u \tag{45}$$

The mean $\langle\delta|t\rangle_G$ is equal to

$$\langle\delta|t\rangle_G = \int_0^{\frac{t\,\langle\mu\rangle_G}{\langle D|t\rangle_G}} \delta\,P(\delta|t)\,\mathrm{d}\delta = \frac{\langle\mu\rangle_G}{\langle D|t\rangle_G}\int_0^t (t-u)P(u)\mathrm{d}u = 1 \tag{46}$$

Substituting Eq. (40) into Eq. (45), the genomic variance of the relative evolutionary rates becomes:

$$\mathrm{Var}(\delta|t) = \langle(\delta|t)^2\rangle_G - \langle\delta|t\rangle_G^2 = \frac{\int_0^t (t-u)^2 P(u)\mathrm{d}u}{\left(\int_0^t (t-u)P(u)\mathrm{d}u\right)^2} - 1 = \frac{\mathrm{Var}(t_{free}|t)}{\langle t_{free}|t\rangle_G^2} \tag{47}$$

where the new variable t_{free} (the free-evolving time) represents the time elapsed since a region escaped the recombination barrier. Therefore, the standard deviation of the relative evolutionary rates is equal to the coefficient of variation of the free-evolving times, which is small if all the genomic regions escaped the recombination barrier a long time ago, and large if parts of the genome are still under the homogenizing effects of homologous recombination.

Supplementary References

1. Cohen, O. & Pupko T. Inference of gain and loss events from phyletic patterns using stochastic mapping and maximum parsimony–a simulation study. *Genome biology and evolution*, **3**, 1265-1275 (2011).

2. Sela, I., Wolf, Y.I. & Koonin, E.V. Theory of prokaryotic genome evolution. *Proc. Natl. Acad. Sci. USA*, **113**, 11399-11407 (2016).

3. Dixit, P.D., Pang, T.Y. & Maslov, S. Recombination-driven genome evolution and stability of bacterial species. *Genetics*, **207**, 281-295 (2017).

4. Iranzo, J., Cuesta, J.A., Manrubia, S.C., Katsnelson, M.I. & Koonin, E.V. Disentangling the effects of selection and loss bias on gene dynamics. *Proc. Natl. Acad. Sci. USA*, **114**, E5616-E5624 (2017).
