# Supplementary material for: Gene gain and loss push prokaryotes beyond the homologous recombination barrier and accelerate genome sequence divergence
Source: Nat Commun. 2019 Nov 26;10:5376. doi: 10.1038/s41467-019-13429-2 (PMC6879757; doi:10.1038/s41467-019-13429-2)
Supplement: Supplementary file 3 — Reporting Summary [file 41467_2019_13429_MOESM3_ESM.pdf]

nature research

Corresponding author(s): Eugene V. Koonin

Last updated by author(s): Jan 22, 2019

Reporting Summary

Nature Research wishes to improve the reproducibility of the work that we publish. This form provides structure for consistency and transparency in reporting. For further information on Nature Research policies, see Authors & Referees and the Editorial Policy Checklist .

Statistics

For all statistical analyses, confirm that the following items are present in the figure legend, table legend, main text, or Methods section.

n/a	Confirmed	
☐	☒	The exact sample size (*n*) for each experimental group/condition, given as a discrete number and unit of measurement
☐	☒	A statement on whether measurements were taken from distinct samples or whether the same sample was measured repeatedly
☐	☒	The statistical test(s) used AND whether they are one- or two-sided<br>*Only common tests should be described solely by name; describe more complex techniques in the Methods section.*
☒	☐	A description of all covariates tested
☒	☐	A description of any assumptions or corrections, such as tests of normality and adjustment for multiple comparisons
☐	☒	A full description of the statistical parameters including central tendency (e.g. means) or other basic estimates (e.g. regression coefficient) AND variation (e.g. standard deviation) or associated estimates of uncertainty (e.g. confidence intervals)
☐	☒	For null hypothesis testing, the test statistic (e.g. *F*, *t*, *r*) with confidence intervals, effect sizes, degrees of freedom and *P* value noted<br>*Give P values as exact values whenever suitable.*
☒	☐	For Bayesian analysis, information on the choice of priors and Markov chain Monte Carlo settings
☒	☐	For hierarchical and complex designs, identification of the appropriate level for tests and full reporting of outcomes
☒	☐	Estimates of effect sizes (e.g. Cohen's *d*, Pearson's *r*), indicating how they were calculated

Our web collection on statistics for biologists contains articles on many of the points above.

Software and code

Policy information about availability of computer code

Data collection	Genomic data and sequence similarity trees were manually downloaded from the Alignable Tight Genomic Clusters (ATGC) database, http://dmk-brain.ecn.uiowa.edu/ATGC/<br>Genome content trees where built with Gloome v1.266, freely available at http://gloome.tau.ac.il/source.php
Data analysis	Tree visualization was done with FigTree v1.4.3, freely available at https://github.com/rambaut/figtree/releases<br>Model fitting, correction of branch lengths, and statistical analysis was carried out with Matlab R2018b, as described in the Methods section.

For manuscripts utilizing custom algorithms or software that are central to the research but not yet described in published literature, software must be made available to editors/reviewers. We strongly encourage code deposition in a community repository (e.g. GitHub). See the Nature Research guidelines for submitting code & software for further information.

Data

Policy information about availability of data

All manuscripts must include a data availability statement. This statement should provide the following information, where applicable:

- Accession codes, unique identifiers, or web links for publicly available datasets
- A list of figures that have associated raw data
- A description of any restrictions on data availability

The datasets that support the findings of this study are available from the Alignable Tight Genomic Clusters (ATGC) database (http://dmk-brain.ecn.uiowa.edu/ATGC/). A data source file containing the data behind all figures is part of the submission and is referred to in the corresponding figure legends.

Field-specific reporting

Please select the one below that is the best fit for your research. If you are not sure, read the appropriate sections before making your selection.

- [x] Life sciences
- [ ] Behavioural & social sciences
- [ ] Ecological, evolutionary & environmental sciences

For a reference copy of the document with all sections, see nature.com/documents/nr-reporting-summary-flat.pdf

Life sciences study design

All studies must disclose on these points even when the disclosure is negative.

Sample size

We selected 35 genomic clusters from the ATGC database that matched the following criteria: i) maximum pairwise tree distance is at least 0.1 substitutions per site, and ii) the phylogenetic tree contains more than two clades, such that pairwise tree distances are centered around more than two typical values. The largest and most diverse cluster (ATGC001), which encompasses E. coli, Shigella, Salmonella, and Enterobacter, was split in two smaller subclusters (one containing Enterobacter, the other Escherichia/Shigella and Salmonella), each of which satisfies the aforementioned conditions. Thus, we analyzed a total of 36 genomic clusters. To facilitate computational analysis, large clusters were subsampled to keep at most 20 representative genomes per cluster.

Data exclusions

Two out of the 36 genomic clusters described above were excluded from the analysis: ATGC108 (Listeria monocytogenes) and ATGC309 (Campylobacter spp.). In the case of ATGC108, the software used to obtain genome composition trees (Gloome) failed to produce a valid output, likely because of lack of convergence of Gloome's internal algorithm. In the case of ATGC309, tree branch lengths from the sequence similarity tree showed limited correlation with branch lengths in the gene content tree, which led to a poor model fit (R-squared = 0.20). Such low value of R-squared constitutes an outlier with respect to all the fits, which have median R-squared equal to 0.8 and 25th and 75th percentiles equal to 0.65 and 0.90, respectively. Model parameters inferred from low quality fits are poorly informative, and therefore we decided to exclude ATGC309 from further analyses.

Replication

Given the method and the dataset, the result of the analysis is not subject to an experimental uncertainty, and therefore replication is not applicable to our study.

Randomization

Because our study did not involve any a priori allocation of subjects (genomic clusters) into experimental groups, randomization was not relevant to our study.

Blinding

Because our study did not involve any a priori allocation of subjects (genomic clusters) into experimental groups, blinding was not relevant to our study.

Reporting for specific materials, systems and methods

We require information from authors about some types of materials, experimental systems and methods used in many studies. Here, indicate whether each material, system or method listed is relevant to your study. If you are not sure if a list item applies to your research, read the appropriate section before selecting a response.

Materials & experimental systems

n/a	Involved in the study	
x		Antibodies
x		Eukaryotic cell lines
x		Palaeontology
x		Animals and other organisms
x		Human research participants
x		Clinical data

Methods

n/a	Involved in the study	
x		ChIP-seq
x		Flow cytometry
x		MRI-based neuroimaging
