# Supplementary material for: Gene gain and loss push prokaryotes beyond the homologous recombination barrier and accelerate genome sequence divergence
Source: Nat Commun. 2019 Nov 26;10:5376. doi: 10.1038/s41467-019-13429-2 (PMC6879757; doi:10.1038/s41467-019-13429-2)
Supplement: Supplementary file 4 — Description of Additional Supplementary Files [file 41467_2019_13429_MOESM4_ESM.pdf]

Description of Additional Supplementary Files

File Name: Supplementary Data 1
Description: The analyzed genomes, model fit parameters and model selection criteria
